# Trait Mindfulness and Social Support Predict Lower Perceived Stress Burden in Patients Undergoing Radiation Therapy

**DOI:** 10.1016/j.adro.2024.101546

**Published:** 2024-06-03

**Authors:** Dylan J. Cooper, Jacob Eckstein, Baho Sidiqi, Zaker H. Rana, Ariana Matarangas, Ashna Shah, Nefia Chacko, Joseph Mancuso, Travis Minutoli, Alana Zinkin, Kirti Sharma, Ria Mehta, Louis Potters, Bhupesh Parashar

**Affiliations:** a Northwell, New Hyde Park, New York; b Department of Radiation Medicine, Northwell Health Cancer Institute, New Hyde Park, New York; c Donald and Barbara Zucker School of Medicine at Hofstra/Northwell Health, Hempstead, New York

## Abstract

**Purpose:**

Cancer diagnosis and treatment, including radiation therapy (RT), cause significant patient stress. Mindfulness and social support have been shown to help manage the psychological effects of cancer treatment. The objective of our study was to determine the sociodemographic and clinical factors associated with stress burden in patients receiving RT.

**Methods and Materials:**

Patients receiving RT for cancer at a single institution were given a 3-section survey to complete during the first on-treatment visit. The survey included the Perceived Stress Scale, Medical Outcomes Study Social Support Survey, and Mindfulness Attention Awareness Scale, which were used to measure stress, social support, and trait mindfulness, respectively. Linear regression analysis was performed to determine associations between perceived stress and age, patient sex, race and ethnicity, treatment intent, disease site, trait mindfulness, and social support. Factors significant in univariable analysis were analyzed with a multivariable analysis.

**Results:**

A total of 93 patients undergoing RT at a tertiary care academic institution were recruited from July to September 2019. Median scores for Perceived Stress Scale, Medical Outcomes Study Social Support Survey, and Mindfulness Attention Awareness Scale were 14.6 (range, 0-31; SD, 6.9), 4.2 (range, 1-5; SD, 1.0), and 5.1 (range, 3.1-6.0; SD, 0.8), respectively. On univariable analysis, mindfulness and social support were associated with decreased stress burden, and female sex and palliative intent were associated with increased stress burden. These factors all maintained significance in multivariable analysis.

**Conclusions:**

These results suggest measures to improve mindfulness and perceived social support, such as mindfulness meditation and psychoeducational approaches, may lessen the stress burden and improve quality of life for patients undergoing RT. Future studies should analyze the longitudinal impact of individual patient characteristics, including patient sex and treatment intent, to better understand their effects on psychological maladjustment during cancer care.

## Introduction

It is estimated 1 in 2 patients diagnosed with cancer experience cancer-related distress, a multifactorial experience that interferes with the patient's ability to cope effectively with cancer, its symptoms, and its treatment.^1^^,^^2^ Cancer-related distress can take on many forms, ranging from negative emotions, eg, fear, sadness, and rumination, to disabling mental health conditions, such as anxiety and depression. Suicide rates in patients with cancer have increased because of the severity of psychological stress in this population.^3^^,^^4^ The psychological sequelae of cancer reduce quality of life and are associated with inferior clinical outcomes, including cancer incidence, disease control, and rates of overall survival.^5^^,^^6^ Psychological stress is also suggested to be an independent risk factor for cancer metastasis in preclinical models.^7^

Patients undergoing radiation therapy (RT) represent a particularly vulnerable population because acute stress can be debilitating in prolonged treatment courses, often leading to premature treatment termination.^8^ The numerous damaging effects of emotional, social, and psychological distress underscore the need for pragmatic, cost-effective ways to relieve the overall stress burden shouldered by these patients.^9^^,^^10^

Trait mindfulness, also known as dispositional mindfulness, is defined as the innate capacity to pay and maintain attention to present-moment experiences with an open, nonjudgmental attitude. Mindfulness has been studied in its ability to relieve stress in several different patient populations, with recent data suggesting mindfulness-based stress reduction practices are noninferior to escitalopram in the treatment of anxiety disorder.^11^ The mental state of mindfulness is the aim of the low-cost practice of mindfulness meditation.

In addition to mindfulness, social support has been shown to be psychologically protective in both stressful situations and psychiatric disorders such as depression and anxiety.^12^ In addition to lower reported levels of stress-related depression and anxiety, subjects who report high perceived social support are less likely to experience physiologic disturbances related to stress such as dysregulated blood pressure, heart rate, and hormonal secretion.^13^, ^14^ To date, trait mindfulness and social support have not been studied together as potential mediators of cancer-related distress in a population of patients receiving RT.

The objective of this study was to determine the sociodemographic and clinical factors associated with stress burden in patients receiving RT. Specifically, we looked at the association between patients’ trait mindfulness, social support, and perceived stress burden in this population.

## Methods and Materials

### Survey distribution

From July to September 2019, following institutional review board approval, patients receiving RT for cancer at a single tertiary care academic institution were given a paper survey to complete during the first on-treatment visit. Survey responses were entered into an online database. Demographic and clinical data were obtained from the electronic medical record and used to identify any potential associations with other variables of interest.

### Survey structure

The survey included the Mindfulness Attention Awareness Scale (MAAS),^12^ Perceived Stress Scale (PSS),^15^ and Medical Outcomes Study Social Support Survey (MOS),^16^ which were used to measure trait mindfulness, stress, and social support, respectively. The survey comprised 44 questions and 3 sections (Supplementary Fig. E1). The questions in each section and their purpose are described in the following paragraphs.

### Trait mindfulness assessment

Trait mindfulness was measured using MAAS,^12^ which has been validated to demonstrate strong psychometric properties, correlating with enhanced self-awareness, mindfulness mediation practice, behavior self-regulation, and positive emotional states. The scale has been validated in multiple languages across populations of students, physicians, and patients, including both meditators and nonmeditators.^17^, ^18^, ^19^ It has also been shown to have appropriate application in examining the role of mindfulness in cancer patients.^20^ For our study, the MAAS section included a 15-item survey with each item scored on a Likert scale from 1 to 6, with 6 indicating greater trait mindfulness. The scores were averaged to give a composite score, with higher scores denoting increased mindfulness. Trait mindfulness was defined with a cutoff of MAAS score of >5.

### Psychological stress assessment

Psychological stress was assessed in the second section of the survey using the PSS-10,^15^ a 10-item self-report measure designed to explore the extent to which daily life situations over the last month are perceived as unpredictable, uncontrollable, and stressful. It includes 2 dimensions: perceived helplessness (eg, how often have you felt that you were on top of things?), consisting of 6 negatively worded items, and perceived self-efficacy (eg, how often have you been able to control irritations in your life?), consisting of 4 positively worded items. Each item is rated on a 5-point Likert-type scale. PSS-10 scores are obtained by first reversing the scores on the 4 positive items, eg, 0 = 4, 1 = 3, and 2 = 2, then, summing across all 10 items, with higher scores indicating higher perceived stress. The PSS-10 has been shown to correlate with measures of perceived helplessness, depression, anxiety, negative affect, and sleep disturbance.^21^ Soria-Reyes et al's^21^ recent study provided validity evidence for PSS-10 in the population with cancer, which identified patients with breast cancer (BrCA) at risk of psychological maladjustment.

### Social support assessment

Perceived social support was surveyed in the final section of the questionnaire using the MOS scale,^16^ which examines social support through an index of 19 items with 3 functional support subscales: emotional/informational support, tangible support, and affectionate support/positive social interaction. The items, eg, assessing how often respondents can count on others to support them in different situations, are rated on a 5-point Likert-type scale. The MOS is one of the most widely used tools for perception of social support, showing good evidence of validity and reliability.^22^^,^^23^ The 19-item MOS index scores were averaged into a composite score between 1 and 5, with 5 indicating greater perceived social support.

### Statistical analysis

Descriptive statistics were used to present survey respondents’ demographics. Continuous variables were expressed using sample medians and categorical variables were expressed as percentages. To determine risk factors for high patient stress, univariable linear regression analysis was performed using PSS scores as the primary outcome. The following variables were analyzed: patient sex, race (coded with White as the reference), ethnicity (coded with not Hispanic/Latino as the reference), age, treatment intent (curative vs palliative), treatment site (coded with prostate cancer as the reference), MAAS score, and MOS score. Treatment intent was determined based on adherence to institutional treatment directives as outlined by departmental clinical guidelines. Variables with significant associations (*P* < .05) were included in a final multivariable analysis. A *P* value of < .05 was considered significant. All statistical analysis was performed using SPSS 21 (IBM, SPSS Inc).

## Results

### Patient characteristics

There were 99 responses, including 93 complete responders without missing data. Demographic and clinical data for the patients included in the study are listed in Table 1. Of the 93 patients, 50 (53.8%) identified as male/men, and 43 (46.2%) identified as female/women. There were no responses for intersex, transfemale, transmale, genderqueer, or other. The median (SD) age was 69 (12.8) years, which ranged from 31 to 99 years. Curative and palliative treatment regimens were prescribed for 83 (89%) and 10 (11%) patients, respectively. Treatment sites included prostate, breast, head and neck, gastrointestinal, brain and spine, gynecologic, lung, palliative bone, and palliative pelvis.

**Table 1:** Baseline characteristics for all survey respondents

	No. of respondents (%)
Sex	
Men	50 (53.8)
Women	43 (46.2)
Race	
White	65 (69.9)
Black	10 (10.8)
Asian	10 (10.8)
Other	8 (8.6)
Ethnicity	
Not Hispanic or Latino	85 (91.4)
Hispanic or Latino	8 (8.6)
Age (y)	
30-49	5 (5.4)
50-59	20 (21.5)
60-69	27 (29.0)
70-79	23 (24.7)
80-89	16 (17.2)
90+	2 (2.2)
Treatment intent	
Curative	83 (89.2)
Palliative	10 (10.8)
Treatment site	
Prostate	26 (28.0)
Breast	22 (23.7)
HN	14 (15.1)
GI	8 (8.6)
CNS	8 (8.6)
GYN	5 (5.4)
Lung	6 (6.5)
Palliative bone	2 (2.2)
Palliative pelvis	2 (2.2)

*Abbreviations*: CNS = brain and spine; GI = gastrointestinal; GYN = gynecologic; HN = head and neck.

### Survey outcomes

Median scores for PSS, MOS, and MAAS were 14.6 (range, 0-31; SD, 6.9), 4.2 (range, 1-5; SD, 1.0), and 5.1 (range, 3.1-6.0; SD, 0.8), respectively. Mean and SD of PSS, MOS, and MAAS results in survey respondents stratified by clinical characteristics are shown in Table 2.

**Table 2:** Mean and SD of the Perceived Stress Scale, Medical Outcomes Study Social Support Survey, and Mindfulness Attention Awareness Scale results in survey respondents

	PSS, mean (SD)	MOS, mean (SD)	MAAS, mean (SD)
Total (N = 93)	14.6 (6.9)	4.2 (1.0)	5.1 (0.8)
Sex			
Male (n = 50)	13.2 (6.5)	4.15 (1.1)	5.1 (0.8)
Female (n = 43)	16.2 (7.2)	4.29 (0.7)	5.1 (0.8)
Race			
White (n = 65)	13.5 (6.7)	4.23 (1.0)	5.2 (0.7)
Black (n = 10)	17.6 (5.3)	4.35 (0.9)	4.8 (1.0)
Asian (n = 10)	16.0 (7.2)	4.14 (0.7)	4.7 (0.7)
Other (n = 8)	18.1 (8.6)	4.01 (0.7)	5.1 (1.0)
Ethnicity			
Not Hispanic or Latino (n = 85)	14.2 (6.9)	4.2 (0.9)	5.1 (0.8)
Hispanic or Latino (n = 8)	18.0 (6.7)	3.8 (1.2)	5.2 (0.9)
Age (y)			
30-49 (n = 5)	14.8 (7.4)	3.9 (0.8)	5.1 (0.6)
50-59 (n = 20)	15.1 (8.5)	4.5 (0.6)	4.8 (0.8)
60-69 (n = 27)	13.3 (6.0)	4.0 (1.1)	5.1 (0.9)
70-79 (n = 23)	16.0 (7.2)	4.2 (1.2)	5.1 (0.8)
80-89 (n = 16)	13.7 (6.4)	4.2 (0.8)	5.1 (0.5)
90+ (n = 2)	17.0 (4.2)	4.9 (0.2)	5.8 (0.3)
Treatment intent			
Curative (n = 83)	14.0 (6.9)	4.2 (1.0)	5.1 (0.8)
Palliative (n = 10)	19.4 (5.0)	4.0 (1.1)	4.8 (0.8)
Treatment site			
Prostate (n = 26)	12.3 (7.4)	4.1 (1.3)	5.0 (0.9)
Breast (n = 22)	15.6 (6.7)	4.3 (0.7)	5.1 (0.6)
HN (n = 14)	13.6 (7.4)	4.3 (0.9)	5.2 (0.8)
GI (n = 8)	17.1 (5.4)	4.3 (1.0)	5.3 (0.6)
CNS (n = 8)	16.6 (7.4)	4.1 (1.1)	4.8 (1.0)
Lung (n = 6)	13.7 (5.5)	4.5 (0.3)	5.0 (0.6)
GYN (n = 5)	12.2 (5.8)	4.2 (0.9)	5.5 (0.5)
Palliative bone (n = 2)	22.5 (0.7)	2.3 (0.0)	5.0 (1.0)
Palliative pelvis (n = 2)	22.5 (0.7)	4.0 (1.5)	4.2 (1.1)

*Abbreviations*: CNS = brain and spine; GI = gastrointestinal; GYN = gynecologic; HN = head and neck; MAAS = Mindfulness Attention Awareness Scale; MOS = Medical Outcomes Study Social Support Survey; PSS = Perceived Stress Scale.

Table 3 presents the results from the univariable and multivariable linear regression models. In univariable analysis, factors that were significantly associated with PSS were sex (β = 3.01; 95% CI [0.20-5.82]; *P* = .036), palliative intent (β = 5.41, 95% CI [0.92-9.90]; *P* = .019), MAAS score (β = −3.88; 95% CI [−5.57 to −2.20]; *P* < .001), and MOS score (β = −2.46; 95% CI [−3.86 to −1.06]; *P* < .001). Univariable model for disease site was found to be not significant (F[5,87] = 1.11; *P* = .360), and disease site was therefore excluded from the multivariable analysis.

**Table 3:** Univariable and multivariable linear regression analyses of factors associated with mental stress

	Univariable analysis		Multivariable analysis	
	β (95% CI)	*P* value	β (95% CI)	*P* value
Sex				
Male	Ref.	Ref.	Ref.	
Female	3.01 (0.20-5.82)	.036*	3.34 (0.96-5.72)	.006*
Race				
White	Ref.	Ref.		
Black	4.15 (−0.45 to 8.74)	.076		
Asian	2.55 (−2.05 to 7.14)	.274		
Other	4.61 (−0.46 to 9.68)	.074		
Ethnicity				
Not Hispanic or Latino	Ref.	Ref.		
Hispanic or Latino	3.75 (−1.30 to 8.81)	.144		
Age (y)	0.00 (−0.11 to 0.12)	.945		
Treatment intent				
Curative	Ref.		Ref.	
Palliative	5.41 (0.92-9.90)	.019*	4.39 (0.55-8.23)	.026*
Treatment site				
Prostate	Ref.			
Breast	3.28 (−0.69 to 7.26)	.104		
HN	1.26 (−3.29 to 5.81)	.582		
GI	4.76 (−0.80 to 10.31)	.092		
CNS	4.26 (−1.30 to 9.81)	.131		
Other	3.27 (−1.23 to 7.68)	.153		
MAAS score	−3.88 (−5.57 to −2.20)	<.001*	−3.16 (−4.74 to −1.58)	<.001*
MOS score	−2.46 (−3.86 to −1.06)	<.001*	−1.94 (−3.20 to −0.68)	.003*

*Abbreviations*: CNS = brain and spine; GI = gastrointestinal; HN = head and neck; MAAS = Mindfulness Attention Awareness Scale; MOS = Medical Outcomes Study Social Support Survey; Ref. = reference.

⁎ Indicates significance.

Multivariable linear regression was used to assess the impact of covariates on perceived stress, and our model was significant (F[4,88] = 11.64; *P* < .001). Palliative intent (β = 4.39; 95% CI [0.55, 8.23]; *P* = .026) and female sex (β = 3.34; 95% CI [0.96, 5.72]; *P =* .006) maintained positive association with PSS, whereas MAAS score (β = −3.16; 95% CI [−4.74 to −1.58]; *P* < .001) and MOS score (β = −1.94; 95% CI [−3.20 to −0.68]; *P* = .003) remained inversely associated with PSS in the multivariable model. A post hoc power analysis was conducted on the primary outcome PSS score using G*Power.^24^ With a 2-sided alpha of .05, Cohen d of 0.42, and a sample size of 93, we achieved 99.9% power to detect group differences.

**Figure 1:**
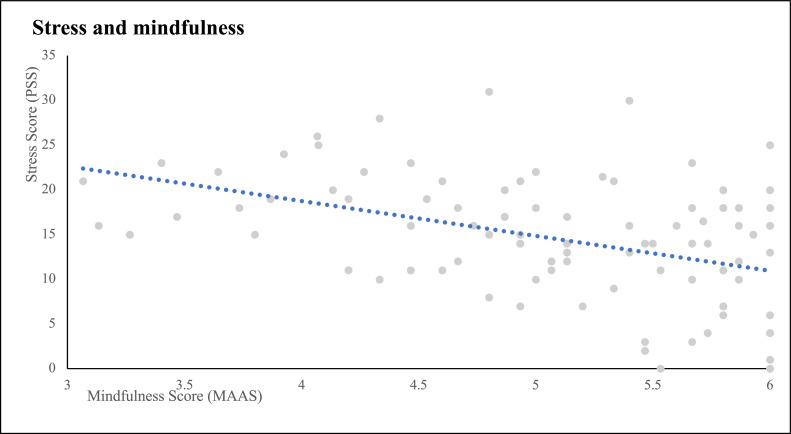
Inverse relationship between stress burden and mindfulness. *Abbreviations*: MAAS = Mindfulness Attention Awareness Scale; PSS = Perceived Stress Scale.

**Figure 2:**
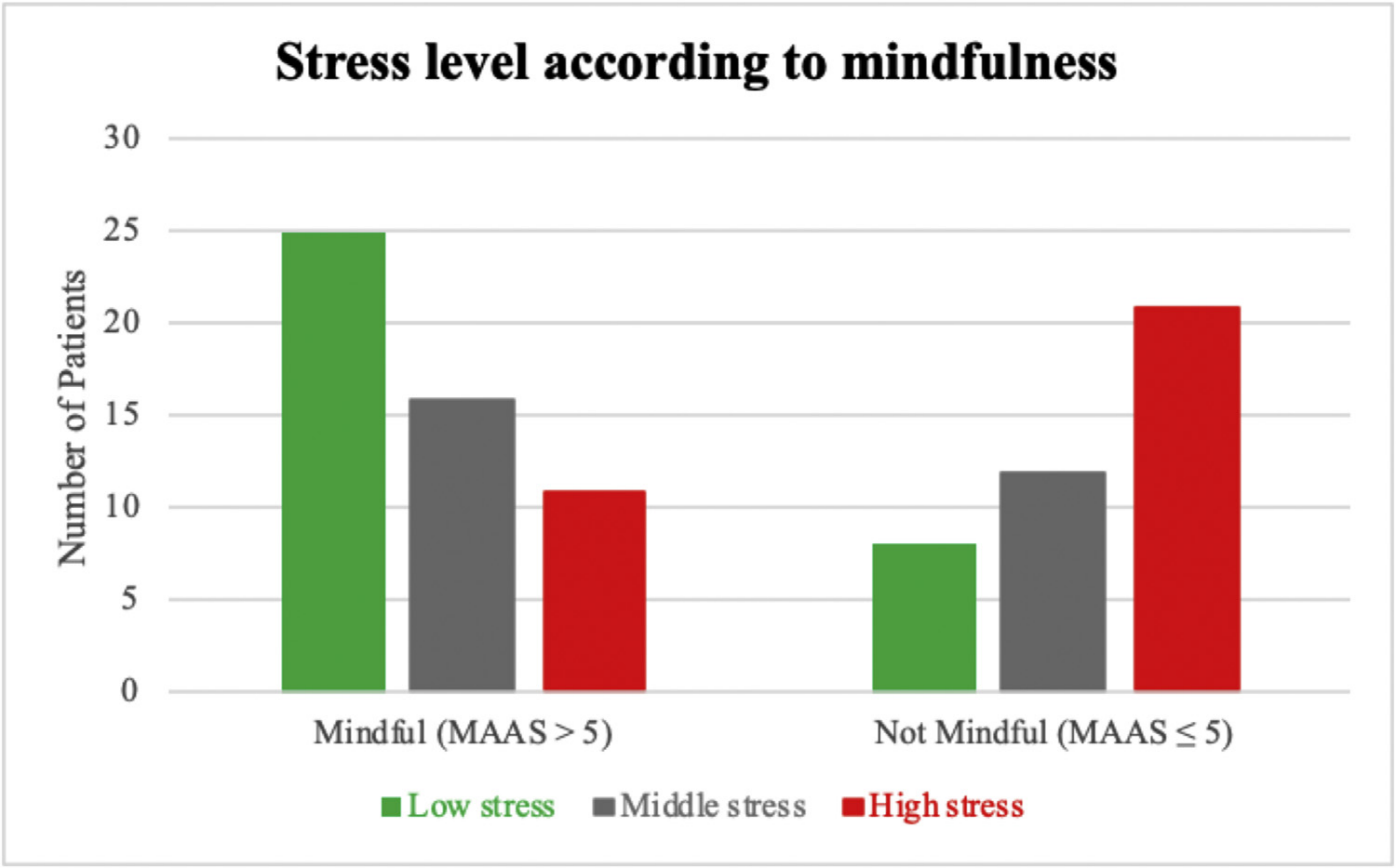
Lower stress levels were found in mindful (Mindfulness Attention Awareness Scale [MAAS] > 5) patients.

The inverse relationship between perceived stress and trait mindfulness (r[93] = −0.433; *P* < .001), seen in this cohort, is shown in Fig. 1. To further characterize this relationship, stress scores were divided into low (PSS < 13), medium (13 ≤ PSS < 18), and high (PSS ≥ 18) categories. Number of scores in the low, medium, and high ranges were 33 (35%), 28 (30%), and 33 (35%) patients, respectively. When a cutoff MAAS score of 5 was applied to discriminate between mindful (MAAS > 5) and not mindful (MAAS ≤ 5) patients, mindful patients were less likely to meet criteria for high stress (PSS ≥ 18), as shown in Fig. 2.

## Discussion

To our knowledge, this is the first cross-sectional study evaluating the interplay of perceived stress, trait mindfulness, and social support in patients receiving RT. In our study, both trait mindfulness and social support were associated with lower stress burden. We also found female sex and palliative intent were associated with higher stress burden in our patient cohort.

### Stress burden

Understanding the landscape of cancer-related distress during RT is a necessary component of patient-focused, comprehensive cancer care because the cancer experience does not occur in a vacuum but in the context of the patient's family, social, and occupational responsibilities, among other ongoing stressors.^25^^,^^26^ The average total PSS score in this patient cohort (14.6; SD, 6.9) compares favorably with similar studies that evaluate stress burden within cancer patients. Golden-Kreutz et al^25^ found an average PSS score in the population with BrCA was 18.3 (SD, 6.96), whereas our cohort's BrCA PSS mean was 15.6 (SD, 6.7). In a 2019 study of 623 cancer survivors of mixed histologies, mean PSS was 17.3.^27^ In a comparative study of 15 active RT patients with cancer and 15 cancer survivors, Ravindran et al^28^ reported a mean PSS of 22.13 in the group receiving RT versus 9.67 in the survivors.

### Trait mindfulness

Although this study is the first, to our knowledge, to investigate trait mindfulness as a negative predictor of stress burden in a population of patients receiving RT, other studies have investigated the impact of mindfulness-based stress reduction programs in cancer care.^29^ Henderson et al^30^ reported a randomized controlled trial in a population of 172 women with newly diagnosed early-stage BrCA receiving RT. They found that a mindfulness-based stress reduction program facilitated reduction in cancer-related anxiety and depression, along with better stress management, improved emotional control, meaningfulness of life, and improved quality of life.^30^, ^31^, ^32^ In addition to the intrinsic value of improving emotional health endpoints, studies have shown that because patients are able to meditate on meaningful, short-term goals, they can develop stress-protective strategies.^31^^,^^33^ In the context of the known benefit of mindfulness meditation in reducing cancer-related distress, our results suggest identification of patients with low trait mindfulness may be a useful heuristic to identify patients with the highest need for intervention.

### Social support

The role of social support in minimizing psychopathologic suffering and cancer-related distress has been reported in the literature.^34^^,^^35^ Social support can be conceptualized as one's perception of informational, emotional, and instrumental support from existing support network members.^36^ It has been found to empower women with BrCA to cope with stress and their disease and to psychologically adapt to cancer-related stressors.^37^^,^^38^ Although the most commonly reported sources of social support are family and friends, obtaining support from one's existing social circle may be challenging at times, and the implementation of social support groups may be an alternative source of support for patients diagnosed and treated for cancer.^39^ Alternatively, development and application of psychoeducational approaches that lead to increased self-esteem and frequency of self-reinforcement have been shown to strengthen perceived social support.^40^ Future studies should further clarify the impact of different sources of social support and psychological sequelae, specifically in patients undergoing RT.

### Female sex

In addition to trait mindfulness and social support, we analyzed the impact of patient sex on perceived stress and found female sex was associated with a higher degree of perceived stress. In previous reports, PSS levels in women have been shown to be higher than those in men.^41^^,^^42^ Meanwhile, in a cross-sectional study in China,^43^ women with newly diagnosed bladder and kidney cancer had numerically higher scores on depression, anxiety, and posttraumatic stress disorder scales than matched men, but these results were not statistically significant. Other reports show conflicting results, demonstrating higher stress levels in men than in women at baseline.^44^ A multi-institutional study^45^ investigating self-questionnaire results of depressive symptoms, perceived social support, and satisfaction with family functioning found no significant differences between female versus male patients with cancer in social support or depressive symptoms. There were also no significant differences by gender in the relationship of network size variables with perceived adequacy or satisfaction with social support.

Although gender roles are changing and an increasing number of men are assuming caregiving roles, the responsibilities of caregiving are traditionally more often carried by women.^46^ Potential factors that may put women at increased risk of cancer-related distress include differences in caretaking support and social support networks among men and women as well as competing household responsibilities.^47^ Regardless of the cause, further investigation is needed to better elucidate the associations between gender and perceived stress in the RT population.

### Palliative intent

Our survey responses revealed patients receiving RT with palliative—rather than curative—intent had a higher stress burden. Other studies have found similar associations between disease and distress severity in cancer cohorts. Peters et al^48^ analyzed 1869 patients and found increased levels of distress in patients with stage IV versus stage III but not stage II or I disease. Kim et al^49^ showed that although psychological distress was common in patients with all stages of gastric cancer, disease stage maintained a statistically significant association with psychological distress. Increased PSS scores in the palliative care cohort may be related to the poor functional status and symptom burden common in this group because fatigue, pain, poor appetite, and low-performance status (Karnofsky Performance Status ≤ 60) can lead to higher PSS scores.^50^

### Cancer type

The association between cancer type and stress burden is more ambiguous. Studies have shown patients with pancreatic cancer and female genital cancers experience the highest levels of distress,^1^^,^^51^ whereas others have found patients with lung cancer to have the highest risk of distress and patients with gynecologic or BrCA to have the lowest rates of distress.^48^^,^^52^ Similar to our findings, Lavelle et al^53^ did not find any significant association between cancer type and cancer-related distress.

### Race and ethnicity

Although statistical significance was not achieved, Black and Hispanic patients showed higher levels of perceived stress burden in our study compared with White and non-Hispanic patients, respectively. This is consistent with the Breast Cancer Care in Chicago study, a population-based, cross-sectional study of 989 patients with newly diagnosed BrCA, which showed that Black and Hispanic patients reported greater PSS compared with White patients and greater unmet social support needs.^54^ Follow-up analyses found socioeconomic position was the root cause of these racial and ethnic disparities.^54^ Other studies have shown that the intersectionality of 2 historically marginalized groups (e.g., Black Americans and women) may result in unique stress experiences for patients, especially Black women.^55^ Longitudinal studies indicate these disparate levels of stress are not isolated to cancer care but may last throughout cancer survivorship as well. Indeed, in one study, White women with BrCA reported less psychological distress from pre- to posttreatment, but distress remained high from diagnosis to 18 months after treatment for their Black counterparts.^56^ Future research is needed to investigate the increased stress burden shouldered by minority groups receiving RT.

### Limitations

Several limitations must be considered when evaluating our findings. This sample comprised patients receiving RT at our tertiary academic center who self-selected into the study, which may impart sampling bias and undermine the external validity of our results.

Further, the small sample size may have resulted in inadequate statistical power to detect some moderate but meaningful differences in the qualitative data, especially among a heterogeneous group of respondents representing multiple disease sites. Similarly, engaging a larger pool of respondents over a longer time period through in-person semistructured interviews may provide a more nuanced account of the experience of perceived stress among patients receiving RT. A limited amount of sociodemographic information was collected, limiting our ability to evaluate the influence of other social identities, such as sexual orientation, educational attainment, economic status, marital status, access to health insurance, and access to care.

Further, we did not ask specific questions about disease stage or details of other treatment choices. Future studies should include more specific measures of disease, cancer staging, and treatment characteristics to better understand the relationship between these factors. Additionally, future studies should include longitudinal data with baseline PSS, MAAS, and MOS scores and serial measurements on follow-up.

## Conclusions

The results of this study highlight the need to consider demographic, treatment-related, and psychosocial variables in managing patients with cancer and cancer-related distress. They also suggest interventions that may enhance mindfulness and social support may be beneficial to patients receiving RT. More studies are necessary to help cancer care providers understand which patients are at highest risk of cancer-related distress so that they can be connected with psychological support as early as possible in the treatment process.

## Disclosures

None.

## Appendix Supplementary materials

Supplemental Figure 1 2-PRO Supplemental Figure 1: Survey distributed to our study cohort, which includes the Mindful Attention Awareness Scale, Perceived Stress Scale, and the Medical Outcomes Social Support Survey. Image, application 1

## Footnotes

Supplementary material associated with this article can be found in the online version at doi:10.1016/j.adro.2024.101546.
