# Supplementary material for: Trait Mindfulness and Social Support Predict Lower Perceived Stress Burden in Patients Undergoing Radiation Therapy
Source: Adv Radiat Oncol. 2024 Jun 3;9(8):101546. doi: 10.1016/j.adro.2024.101546 (PMC11259697; doi:10.1016/j.adro.2024.101546)
Supplement: Supplemental Figure 1 2-PRO — Supplemental Figure 1: Survey distributed to our study cohort, which includes the Mindful Attention Awareness Scale, Perceived Stress Scale, and the Medical Outcomes Social Support Survey. [file mmc1.pdf]

The Mindful Attention Awareness Scale (MAAS)

The trait MAAS is a 15-item scale designed to assess a core characteristic of mindfulness, namely, a receptive state of mind in which attention, informed by a sensitive awareness of what is occurring in the present, simply observes what is taking place.

Brown, K.W. & Ryan, R.M. (2003). The benefits of being present: Mindfulness and its role in psychological well-being. *Journal of Personality and Social Psychology, 84,* 822-848.

Carlson, L.E. & Brown, K.W. (2005). Validation of the Mindful Attention Awareness Scale in a cancer population. *Journal of Psychosomatic Research, 58,* 29-33.

Instructions: Below is a collection of statements about your everyday experience. Using the 1-6 scale below, please indicate how frequently or infrequently you currently have each experience. Please answer according to what really reflects your experience rather than what you think your experience should be. Please treat each item separately from every other item.

1	2	3	4	5	6
almost always	very frequently	somewhat frequently	somewhat infrequently	very infrequently	almost never

_____ 1. I could be experiencing some emotion and not be conscious of it until some time later.
_____ 2. I break or spill things because of carelessness, not paying attention, or thinking of something else.
_____ 3. I find it difficult to stay focused on what’s happening in the present.
_____ 4. I tend to walk quickly to get where I’m going without paying attention to what I experience along the way.
_____ 5. I tend not to notice feelings of physical tension or discomfort until they really grab my attention.
_____ 6. I forget a person’s name almost as soon as I’ve been told it for the first time.
_____ 7. It seems I am “running on automatic,” without much awareness of what I’m doing.
_____ 8. I rush through activities without being really attentive to them.
_____ 9. I get so focused on the goal I want to achieve that I lose touch with what I’m doing right now to get there.
_____ 10. I do jobs or tasks automatically, without being aware of what I'm doing.
_____ 11. I find myself listening to someone with one ear, doing something else at the same time.
_____ 12. I drive places on ‘automatic pilot’ and then wonder why I went there.
_____ 13. I find myself preoccupied with the future or the past.
_____ 14. I find myself doing things without paying attention.
_____ 15. I snack without being aware that I’m eating.

Scoring: To score the scale, simply compute a mean (average) of the 15 items.

PSS

INSTRUCTIONS:

The questions in this scale ask you about your feelings and thoughts during THE LAST MONTH. In each case, please indicate your response by placing an "X" over the circle representing HOW OFTEN you felt or thought a certain way.

	Never<br>0	Almost Never<br>1	Sometimes<br>2	Fairly Often<br>3	Very Often<br>4
**1. In the last month, how often have you been upset because of something that happened unexpectedly?**	○	○	○	○	○
**2. In the last month, how often have you felt that you were unable to control the important things in your life?**	○	○	○	○	○
**3. In the last month, how often have you felt nervous and "stressed"?**	○	○	○	○	○
**4. In the last month, how often have you felt confident about your ability to handle your personal problems?**	○	○	○	○	○
**5. In the last month, how often have you felt that things were going your way?**	○	○	○	○	○
**6. In the last month, how often have you found that you could not cope with all the things that you had to do?**	○	○	○	○	○
**7. In the last month, how often have you been able to control irritations in your life?**	○	○	○	○	○
**8. In the last month, how often have you felt that you were on top of things?**	○	○	○	○	○
**9. In the last month, how often have you been angered because of things that were outside your control?**	○	○	○	○	○
**10. In the last month, how often have you felt difficulties were piling up so high that you could not overcome them?**	○	○	○	○	○

THE Medical Outcomes Survey (MOS) SOCIAL SUPPORT SURVEY

People sometime s look to others for companionship, assistance, or other types of support. How often is e ach of the following kinds of support available to you if you need it?
Circle one number on each line .

	None of the time	A little of the time	Some of the time	Most of the time	All of the time
**Emotional/informational support**					
Someone you can count on to listen to you when you need to talk	1	2	3	4	5
Someone to give you information to help you understand a situation	1	2	3	4	5
Someone to give you good advice about a crisis	1	2	3	4	5
Someone to confide in or talk to about yourself or your problems	1	2	3	4	5
Someone whose advice you really want	1	2	3	4	5
Someone to share your most private worries and fears with	1	2	3	4	5
Someone to turn to for suggestions about how to deal with a personal problem	1	2	3	4	5
Someone who understands your problems	1	2	3	4	5
**Tangible support**					
Someone to help you if you were confined to bed	1	2	3	4	5
Someone to take you to the doctor if you needed it	1	2	3	4	5
Someone to prepare your meals if you were unable to do it yourself	1	2	3	4	5
Someone to help with daily chore s if you were sick	1	2	3	4	5
**Affectionate support**					
Someone who shows you love and affection	1	2	3	4	5
Someone to love and make you feel wanted	1	2	3	4	5
Someone who hugs you	1	2	3	4	5
**Positive social interaction**					
Someone to have a good time with	1	2	3	4	5
Someone to get together with for relaxation	1	2	3	4	5
Someone to do something enjoyable with	1	2	3	4	5
**Additional item**					
Someone to do things with to help you get your mind off things	1	2	3	4	5
